# State-of-the-Art Dental Adhesives and Restorative Composites

**DOI:** 10.3390/jfb17080412

**Published:** 2026-08-18

**Authors:** Jirun Sun

**Affiliations:** ADA Forsyth Institute, Somerville, MA 02143, USA; jsun@forsyth.org

Since the pioneering work of Dr. Rafael Bowen in the early 1960s [1,2], methacrylate-based resin composites have fundamentally transformed restorative dentistry [3,4,5]. Over the ensuing decades, these materials have evolved into the dominant choice for dental restorations, owing to their favorable balance of esthetics, adhesion, and mechanical performance. Despite this success, continued improvements in durability, interfacial stability, and functional performance remain central challenges, driving ongoing innovation in dental materials science.

Contemporary dental adhesives and restorative composites are complex, multi-component systems typically consisting of a polymerizable resin matrix, reinforcing filler particles, and functional additives. The performance of these materials is governed by the interplay among polymer network structure, filler–matrix interactions, and the physicochemical environment of the oral cavity. Subtle variations in monomer chemistry, degree of conversion, crosslink density, and filler surface treatment can significantly influence mechanical properties, water sorption, degradation kinetics, and interfacial behavior. As such, the rational design of these materials increasingly relies on a deeper understanding of structure–property–function relationships.

This Special Issue, “State-of-the-Art Dental Adhesives and Restorative Composites”, reflects these themes by bringing together contributions that span polymer chemistry, interfacial science, and clinically relevant material performance. The selected works collectively illustrate how advances at the molecular and microstructural levels translate into improved functional outcomes.

A key direction highlighted in this collection is the incorporation of functional and bioactive components into polymer networks. Almutairi et al. demonstrate that embedding the quaternary ammonium monomer dimethylaminohexadecyl methacrylate (DMAHDM) into a UDMA/TEG-DVBE resin system can yield substantial antibacterial activity against *Streptococcus mutans* biofilms while preserving mechanical properties [6]. This work underscores how molecular-level design—specifically, the integration of polymerizable antimicrobial moieties—can enable sustained functionality without compromising network integrity. Such strategies illustrate the broader potential of designing polymer networks that couple mechanical performance with targeted biological activity.

The evolution of composite materials is further contextualized by Ferracane’s historical perspective, which outlines how advances in filler technology, photopolymerization, and rheological control have enabled improvements in strength, wear resistance, and handling [5]. Importantly, this progression reflects a continuous refinement of composite microstructure, including optimization of filler size distribution, loading, and surface chemistry to enhance stress transfer and minimize defects within the polymer matrix.

Interfacial degradation remains a critical limitation in adhesive dentistry, and Pfeifer et al. provide a material-focused analysis of the mechanisms involved [7]. Hybrid layer degradation is driven by a combination of enzymatic activity and hydrolytic instability of the polymer network, particularly in hydrophilic adhesive formulations. Emerging material strategies—including the development of ester-free or acrylamide-based polymers, incorporation of enzyme inhibitors, and reduction in water sorption—highlight the importance of designing chemically stable networks with improved resistance to environmental degradation. These approaches emphasize the need to control both polymer chemistry and interfacial architecture to achieve durable adhesion.

Advances in restorative materials are also closely linked to processing technologies. Ille et al. review chairside CAD/CAM materials, where the relationship between composition, microstructure, and machinability becomes particularly important [8]. Materials such as lithium disilicate, zirconia, and hybrid ceramics must balance mechanical strength with fracture resistance and processability. The increasing use of digitally fabricated restorations underscores the need for materials with well-controlled microstructures that can withstand both machining and long-term functional loading.

At the level of polymer network design, Salazar et al. explore the use of novel tetraurethane diacrylates (TUDAs) to achieve enhanced mechanical performance [9]. Their work highlights how monomer architecture and secondary (non-covalent) interactions can influence polymerization kinetics and network formation, leading to improved mechanical properties. At the same time, the observed sensitivity to aqueous environments underscores the persistent challenge of designing networks that maintain performance under clinically relevant conditions. This study exemplifies how iterative formulation and structure–property analysis are essential for optimizing composite materials.

Collectively, the contributions in this Special Issue underscore several important materials-science themes: the design of functional polymer networks, control of filler–matrix interactions and composite microstructure, mitigation of hydrolytic and enzymatic degradation, and integration of materials with advanced processing technologies. These directions reflect a broader shift toward more predictive and mechanism-driven approaches to dental material development.

Looking ahead, progress in dental adhesives and restorative composites will likely depend on further elucidation of structure–property relationships across multiple length scales, from molecular interactions to bulk composite behavior. Improved control over polymer network architecture—including crosslink density, heterogeneity, and dynamic interactions—may enable materials with enhanced toughness and resistance to degradation. In addition, advances in surface chemistry and interfacial engineering are expected to play a critical role in improving adhesion durability. While emerging approaches such as data-driven formulation and responsive materials offer new opportunities, their impact will depend on the ability to translate these concepts into materials with consistent, reproducible performance under oral conditions. Continued integration of advanced characterization techniques and clinically relevant testing models will be essential to guide these developments.

In summary, this Special Issue highlights how continued innovation in materials chemistry, microstructure design, and interfacial engineering is advancing the performance of dental adhesives and restorative composites. By linking fundamental materials science with clinical requirements, these efforts contribute to the development of restorative systems capable of delivering improved durability and function in the complex oral environment.

## Data Availability

No new data were created or analyzed in this study. Data sharing is not applicable to this article.
